# Endoscopic ultrasound navigated application of botulinum toxin in severe esophageal motility disorder

**DOI:** 10.1007/s12328-024-02066-y

**Published:** 2024-12-03

**Authors:** Diana Vážanová, Martin Ďuriček, Peter Uhrík, Peter Bánovčin

**Affiliations:** Clinic of Internal Medicine–Gastroenterology, University Hospital in Martin, Jessenius Faculty of Medicine, Martin, Slovakia

**Keywords:** Botulinum toxin, Endoscopic ultrasound, Motility disorder of esophagus

## Abstract

The use of botulinum toxin in the therapy of esophageal motility disorders is reserved for elderly and comorbid patients considered risky for endoscopic or surgical treatment. However, there is a lack of data on the treatment of motility disorders outside the Chicago classification.

We present the case of a 56-year-old patient with dysphagia and non-cardial chest pain (Eckardt 8). High resolution manometry ruled out achalasia or other motility disorder, but confirmed a localized 7-cm-long spastic segment in the upper to middle third of esophagus. We considered endoscopic or surgical therapy in this location too risky, therefore we decided to apply botulinum toxin into this segment. The spasm on high resolution manometry correlated with the thickened muscularis propria layer according to the endoscopic ultrasound. We used endoscopic ultrasound for the navigation of botulinum toxin application into the muscularis propria layer. We applied 100 IU of botulinum toxin into four quadrants, 20 and 24 cm from front teeth (12.5 IU for 1 application).

The therapy led to improvement of symptoms (Eckardt 3) and to restitution of propulsive peristalsis with complete elimination of spastic segment. The worsening of symptoms appeared after 2 years, with subsequent recurrence of motility disorder fulfilling criteria of type II achalasia.

Presenting this case, we wanted to point at the unique use of botulinum toxin as useful treatment in selected cases of unclassified esophageal motility disorder as a bridge therapy. Moreover, endoscopic ultrasound could be used to guide precise application of botulinum toxin.

## Introduction

The role of botulinum toxin in the treatment of esophageal motility disorders has been repeatedly discussed [1], although without any encouraging recommendation for its routine use [2–4]. The most researched role was in patients with achalasia [5], where the botulinum toxin is applied into the lower esophageal sphincter (LES). The level of evidence in the non-achalasia esophageal motility disorders is somewhat scarce. The landmark study by Vanuytsel et al. confirmed the effect of botulinum toxin injected in the esophageal body in the improvement of dysphagia and reversal of weight loss one month after the application in patients with distal esophageal spasm (DES) and hypercontractile esophagus (HE) [6]. Conversely, botulinum toxin did not show any superior role to saline injection regarding non cardiac chest pain, heartburn, and regurgitation. Here we present a case report describing the endoscopic ultrasound-navigated application of botulinum toxin in severely symptomatic patient with unclassified esophageal motility disorder. To the best of our knowledge, this is the first use of EUS navigation of botulinum toxin application into the esophagus in its motility disorder.

## Case report

**Fig. 1:**
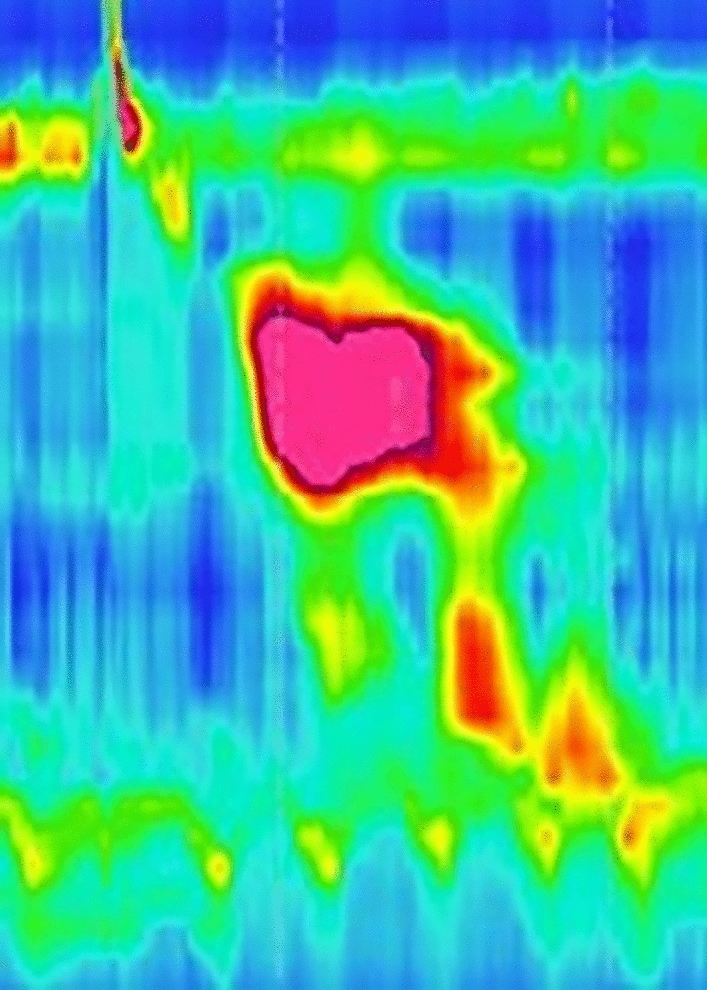
HRM study from the first visit of patient, where the relaxation pressure of lower esophageal sphincter was in physiological range (IRP 10,4 mmHg) with a 7 cm long spastic segment from 23 to 30 cm from nostrils

**Fig. 2:**
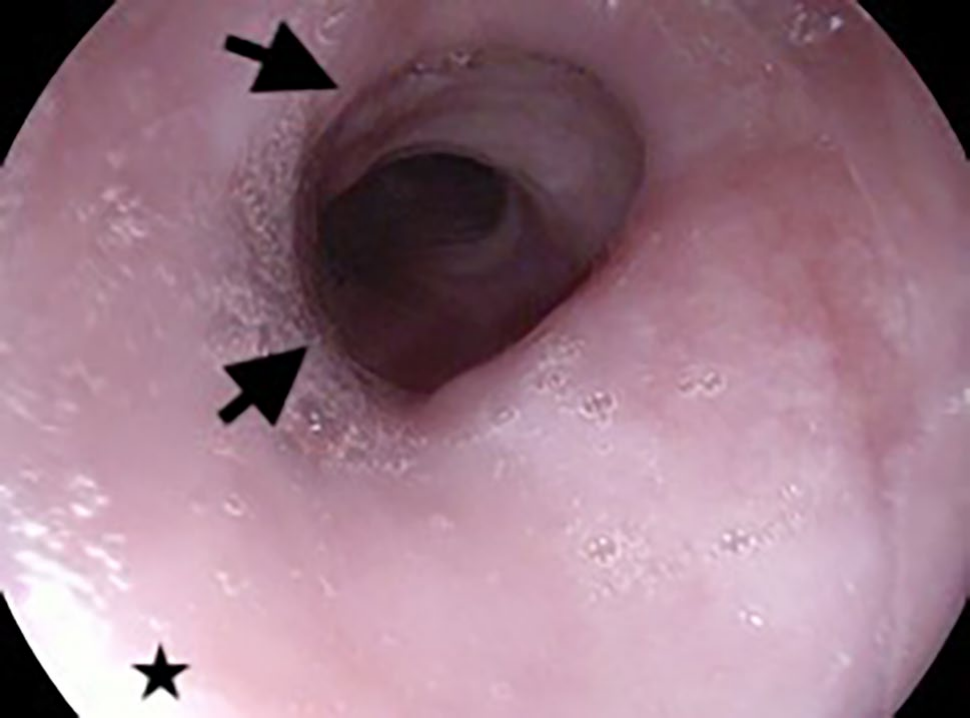
Upper endoscopy with no stenosis of the lumen and pseudodiverticular dilatation in the upper third of the esophagus, 2 arrows point at spastic segment and a star points at pseudodiverticulum

We state that subject of this case report gave written informed consent to publish her case, including publication of images. A 56-year-old female patient was recommended for evaluation to our tertiary center/motility laboratory due to dysphagia for solids and liquids, non-cardiac chest pain related to eating that had been lasting for 4 months and regurgitation with no effect of proton pump inhibitors. No weight loss was reported. Eckardt score was 8. Her past medical history included arterial hypertension, alcohol use disorder, and depressive disorder. Her medical history included proton pump inhibitors, hypotensive medication, venlafaxine, mirtazapine, and alprazolam**.** Importantly, the patient reported no opiate use. High-resolution manometry (HRM) showed normal LES integrated relaxation pressure (IRP 10.4 mmHg), signs of propulsive peristalsis and no panesophageal pressurization (Fig. 1), however, it revealed a 7 cm long spastic segment extending from 23 to 30 cm from the nostrils (maximum pressure of 322 mmHg during the motility study within this segment), elevated distal contractile interval (DCI, maximum of 7762 mmHg in a single swallow, mean value 5482 mmHg). The spastic segment (Fig. 1) was in manometric study clear in 80% of 5 ml swallows in the upright position. Distal latency (DL) was normal (8.9 s). Neither achalasia, nor any other spastic motility disorder could have been confirmed. The barium swallow study showed free passing of the contrast agent into the stomach. Due to inconclusive results, we proceeded to contrast CT scan that revealed esophageal wall thickening of 8 mm in the proximal part (maximal thickness of 15 mm) corresponding to the spastic segment revealed by HRM. While performing investigations, we administered nitroglycerine 15 min. before main dishes as an empirical therapy with limited symptomatic effect. We performed upper endoscopy (Fig. 2) that revealed no narrowing of the lumen however there was pseudodiverticular dilatation of esophageal lumen and tertiary spasms below. We repeated HRM to exclude the development of achalasia or Jackhammer esophagus, but its finding was almost unchanged, (IRP slightly elevated − 18.8 mmHg with signs of propulsive peristalsis, spastic segment still present, mean DCI 2903 mmHg and DL 8.6 s).

**Fig. 3:**
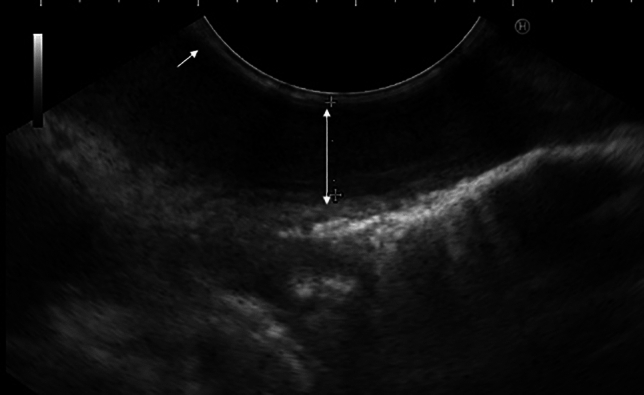
EUS image of thickened muscular layer of the upper third of the esophagus, white two-sided arrow points at muscularis propria, white arrow points at mucosal and submucosal layer represented by hyperechogenic and hypoechogenic line between the muscularis propria and the probe

**Fig. 4:**
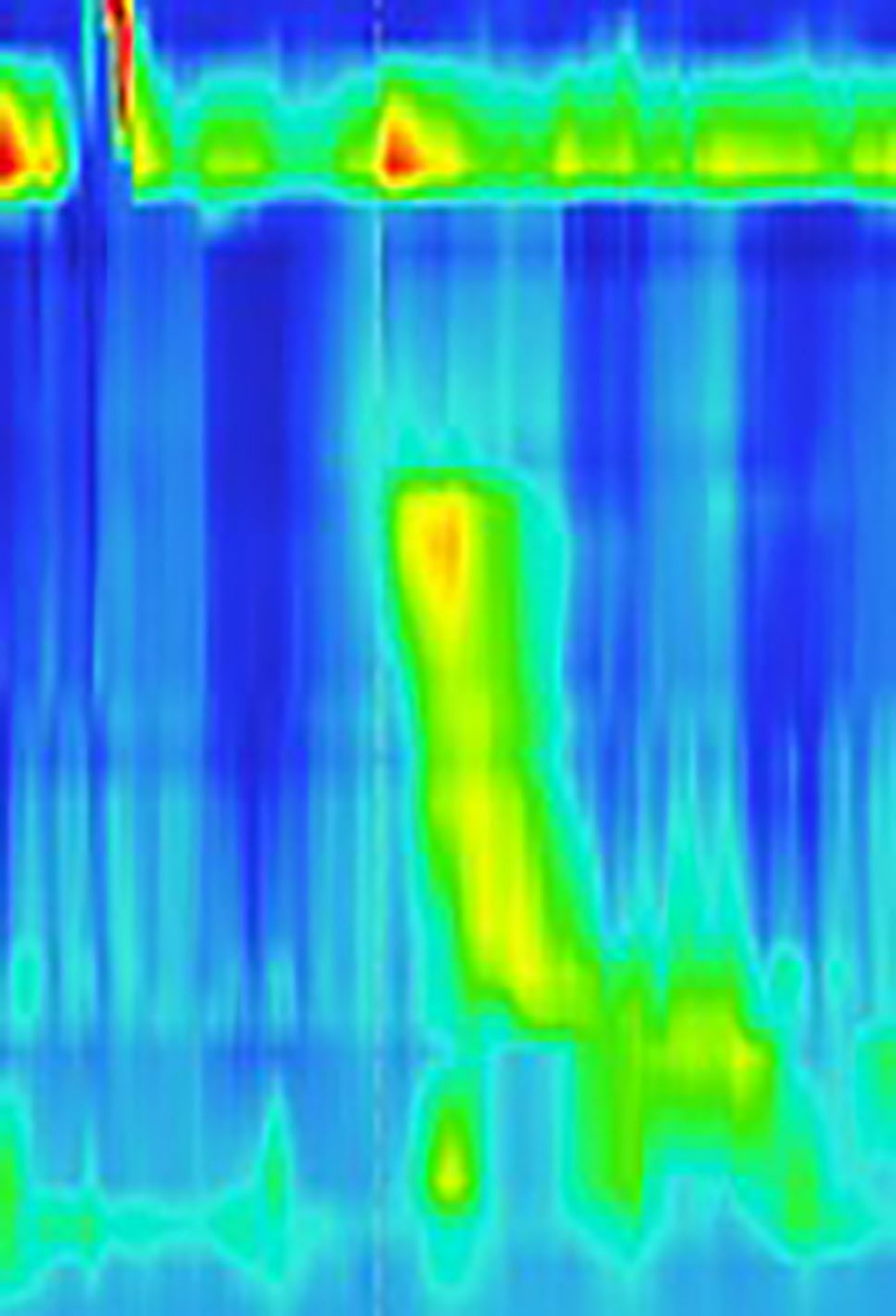
HRM study 3 weeks after the botulinum toxin application and restoration of the propulsive peristalsis, normal LES relaxation pressure

**Fig. 5:**
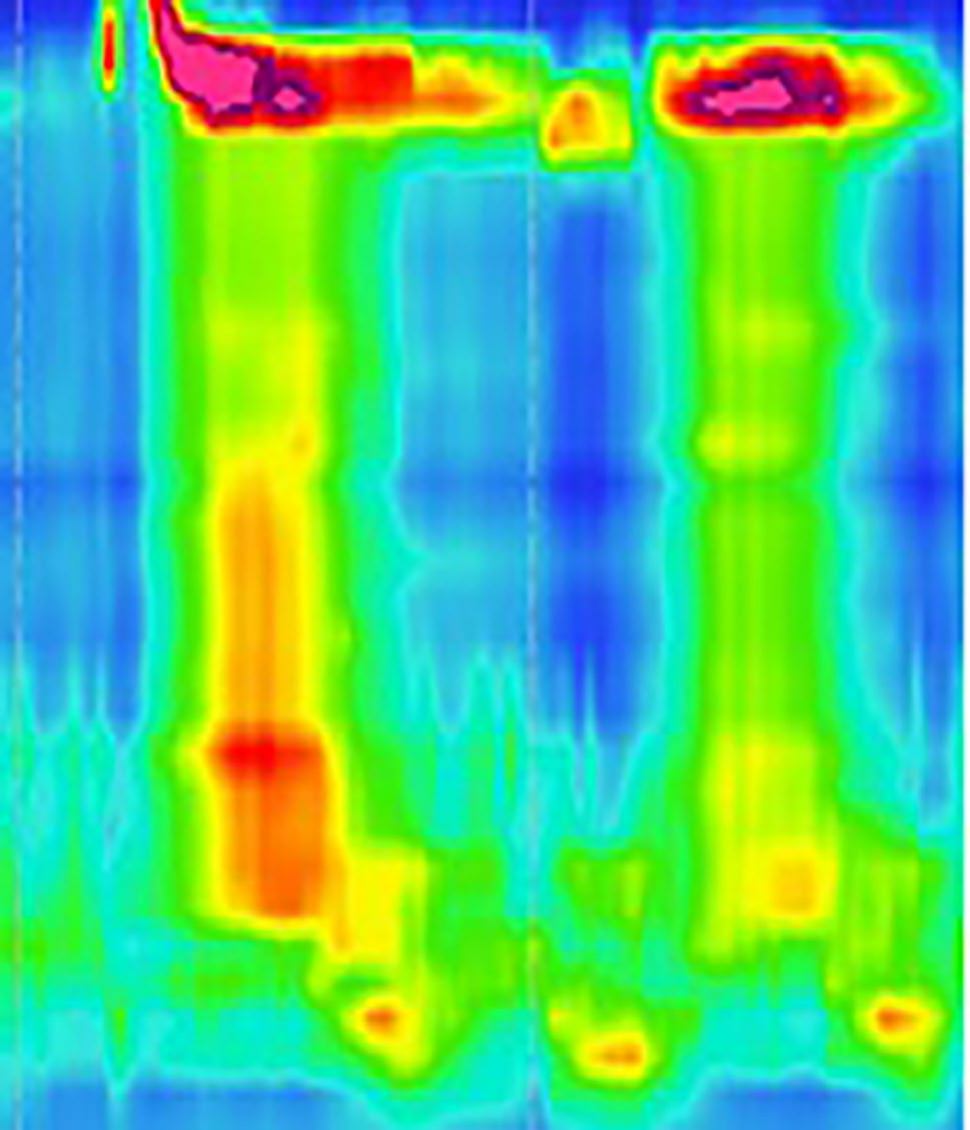
HRM image with panesophageal pressurization and elevated LES relaxation pressure suggesting type II. Achalasia (IRP 21 mmHg). *HRM* high resolution manometry, *LES* lower esophageal sphincter, *IRP* integrated relaxation pressure

Considering high symptom burden and not yet classifiable motility disorder we decided to apply botulinum toxin into the esophageal body. To secure its strict application into the bounded segment of thickened muscle layer of the esophagus (that was believed to be the major cause of symptoms) we decided to use EUS (endoscopic ultrasound) navigation. Because of the EUS probe compression, the submucosal and mucosal layer are visible only as a thin hyperechogenic and hypoechogenic lines between probe and muscularis propria (Fig. 3). After localizing the thickened part of the esophageal wall (20–30 cm from front teeth according to the EUS), we applied botulinum toxin into 4 quadrants at the level of 24 cm from front teeth (muscularis propria layer thickness 8.8 mm) (Fig. 3) and 20 cm from front teeth (muscularis propria layer thickness 4.9 mm). Botox Allergan (Allergan Pharmaceuticals Ireland) was dissolved in 8 ml of sterile saline solution, resulting in 12.5 IU/1 ml for the single application. Indeed, EUS-FNI (endoscopic ultrasound–fine needle injection) technology was employed with the use of the Olympus MAJ-67 needle. At the checkup 3 weeks after the procedure the patient reported improvement of symptoms and restoration of propulsive peristalsis on HRM with total elimination of spastic segment (Fig. 4). The Eckardt score was 3. Gradual worsening of symptoms occurred 2 years after botulinum toxin injection, with Eckhardt score 8 (03/2021). The COVID-19 pandemic postponed scheduled visits of the patient and as late as in 05/2022 during another follow-up visit HRM revealed features fulfilling the diagnosis of II type achalasia (Fig. 5), with panesophageal pressurization in 40% of swallows, IRP was 21 mmHg. As the diagnosis of achalasia based on the HR manometry image was clear, we did not further confirm it with esophagogram and proposed peroral endoscopic myotomy to the patient.

## Discussion

Our case report describes the use of EUS to guide intramuscular application of the botulinum toxin in patient with yet unclassified esophageal motility disorder**.**

The therapeutic potential for botulinum toxin in esophageal motility disorders has been first described in achalasia patients [7]. Its time-limited effect has been demonstrated in multiple studies [8–10]. This restricted its utilization for elderly and/or comorbid patients in whom no requirement for anesthesia and low incidence of undesirable effects outnumbers the transient effect [1, 9–11]. In achalasia, it is applied into the LES region, while in distal esophageal spasm and Jackhammer esophagus it is injected into the esophageal body. For all above mentioned indications, botulinum toxin is applied endoscopically into the muscularis propria layer.

Some significant landmarks in the area of botulinum toxin utilization in esophageal motility disorders could be highlighted based on our case. From the motility point of view, the possibility to apply botulinum toxin in a disorder with spastic component that is not yet classified according to the Chicago classification. Although one might argue that during the second visit of the patient the manometric finding was esophageal outflow obstruction, we considered the spasm in the mid esophagus more relevant for the symptoms, and therefore, a target for therapy. Significant symptom improvement further supports our approach. From the clinical point of view, there is the potential to expand the indications for the use of botulinum toxin not only in elderly comorbid patients, but also for younger subjects with yet unspecified motility defect. As it is shown by the natural course of the disease, this approach led to substantial symptom improvement until the motility disorder developed into achalasia.

Last but not least, our case points out the possibility of significantly more precise EUS navigation of botulinum toxin application rather than more common endoscopic navigation. Although the use of EUS is in the esophagus, it has been mostly indicated for sampling lymph nodes or metastases in neoplastic lesions [12]. EUS-guided tissue acquisition (TA) using fine needle aspiration (FNA) or fine needle biopsy (FNB) has been also widely used for subepithelial lesions (originating in the submucosa or muscularis propria) [13]. We took the advantage of the fact that the muscle layer was significantly thickened in our patient in the level of the spastic segment (similarly to thickened muscle at the level of the lower esophageal sphincter in patients with achalasia) [14]. Our approach might prevent complications (e. g. mediastinitis due to paraesophageal application) not only in unclassified motility disorders, but also patients with distal esophageal spasm or Jackhammer esophagus that are more commonly indicated for botulinum toxin application.

The relief of symptoms of our patient was considerable. Importantly, the effect was also confirmed by HRM with complete (although transient) elimination of the spastic segment that limits the possibility of the placebo effect.

## Conclusion

We suggest that in selected cases botulinum toxin injection might be used in motility disorders outside the Chicago classification, possibly with EUS navigation securing accurate application. This approach allows to gain time while the motility disorder is not yet developed.
